# Supplementary material for: Unlocking insights: Mahout’s perceptions and practices in managing Elephant Endotheliotropic Herpesvirus (EEHV) infection among captive Asian elephants in Surin province, Thailand
Source: PLoS One. 2024 Nov 12;19(11):e0295869. doi: 10.1371/journal.pone.0295869 (PMC11556736; doi:10.1371/journal.pone.0295869)
Supplement: S1 Dataset — (DOCX) [file pone.0295869.s001.docx]

**Raw data used for determining means, standard deviations, and comparative analysis that showed in Table 1-6.**

| **Ma**  **hout** | **experiance** | **ele**  **no.** | **age** | **Cal**  **adu** | **k.**  **sign** | **k.**  **trans** | **k.**  **prac** | **k.**  **sum** | **child** | **death** | **cure** | **new**  **ele** | **trans** | **wean** | **Vit** | **E**  **E**  **H**  **V** | **a.**  **sum** | **De**  **worm** | **food** | **stall** | **exercise** | **sum**  **norm** | **Non**  **contact**  **ele** | **human**  **non** | **no**  **con**  **sick** | **obse** | **sum**  **spec** | **p.**  **sum** |
| --- | --- | --- | --- | --- | --- | --- | --- | --- | --- | --- | --- | --- | --- | --- | --- | --- | --- | --- | --- | --- | --- | --- | --- | --- | --- | --- | --- | --- |
| **1** | 38 | 1 | 55 | a | 1 | 0 | 0 | 1 | 2 | 2 | 1 | 2 | 2 | 2 | 1 | 1 | 13 | 1 |  |  |  | 1 |  |  |  |  | 0 | 1 |
| **2** | 28 | 2 | 43 | a | 0 | 0 | 0 | 0 | 0 | 2 | 1 | 2 | 1 | 1 | 1 | 2 | 12 | 1 |  |  |  | 1 |  |  | 1 |  | 1 | 2 |
| **3** | 21 | 2 | 33 | a | 4 | 6 | 3 | 13 | 2 | 2 | 1 | 1 | 2 | 1 | 2 | 0 | 11 | 0 |  |  | 1 | 1 | 1 |  |  |  | 1 | 2 |
| **4** | 33 | 2 | 46 | a | 2 | 0 | 0 | 2 | 2 | 2 | 2 | 2 | 2 | 2 | 1 | 1 | 14 | 1 | 1 | 1 |  | 3 |  |  |  |  | 0 | 3 |
| **5** | 35 | 1 | 47 | a | 2 | 5 | 0 | 7 | 2 | 2 | 2 | 2 | 2 | 2 | 2 | 1 | 15 | 0 | 1 |  |  | 1 |  |  |  |  | 0 | 1 |
| **6** | 43 | 1 | 55 | a | 1 | 3 | 0 | 4 | 2 | 0 | 0 | 0 | 0 | 0 | 0 | 0 | 0 | 1 | 1 |  |  | 2 |  |  | 1 |  | 1 | 3 |
| **7** | 32 | 3 | 44 | a | 2 | 0 | 2 | 4 | 2 | 1 | 2 | 2 | 2 | 0 | 2 | 1 | 12 | 0 |  |  |  | 0 | 1 |  |  |  | 1 | 1 |
| **8** | 12 | 1 | 24 | a | 1 | 3 | 0 | 4 | 2 | 2 | 2 | 2 | 2 | 1 | 0 | 0 | 11 | 1 | 1 |  |  | 2 |  |  |  |  | 0 | 2 |
| **9** | 12 | 1 | 25 | a | 3 | 0 | 0 | 3 | 2 | 2 | 2 | 2 | 2 | 2 | 1 | 3 | 16 | 1 |  | 1 |  | 2 |  |  |  |  | 0 | 2 |
| **10** | 63 | 3 | 63 | a | 0 | 0 | 2 | 2 | 0 | 2 | 2 | 2 | 2 | 2 | 2 | 3 | 17 | 1 |  | 1 | 1 | 3 | 1 |  |  |  | 1 | 4 |
| **11** | 33 | 1 | 48 | a | 1 | 0 | 0 | 1 | 0 | 1 | 2 | 2 | 2 | 2 | 2 | 2 | 15 | 1 |  | 1 |  | 2 |  |  |  |  | 0 | 2 |
| **12** | 17 | 2 | 38 | a | 2 | 0 | 1 | 3 | 2 | 2 | 2 | 0 | 2 | 2 | 1 | 0 | 11 | 0 |  |  |  | 0 |  |  |  |  | 0 | 0 |
| **13** | 20 | 1 | 36 | a | 2 | 0 | 0 | 2 | 0 | 2 | 0 | 0 | 2 | 2 | 2 | 2 | 12 | 1 |  | 1 |  | 2 |  |  |  |  | 0 | 2 |
| **14** | 2 | 7 | 14 | a | 3 | 6 | 1 | 10 | 2 | 2 | 0 | 0 | 2 | 2 | 2 | 3 | 13 | 1 |  |  |  | 1 | 1 |  |  |  | 1 | 2 |
| **15** | 25 | 5 | 37 | a | 3 | 3 | 3 | 9 | 2 | 2 | 2 | 2 | 2 | 2 | 1 | 1 | 14 | 0 |  | 1 |  | 1 | 1 |  |  |  | 1 | 2 |
| **16** | 25 | 8 | 38 | a | 2 | 0 | 3 | 5 | 2 | 2 | 2 | 1 | 2 | 0 | 2 | 2 | 13 | 1 |  |  |  | 1 |  |  |  | 1 | 1 | 2 |
| **17** | 38 | 6 | 50 | a | 3 | 3 | 0 | 6 | 2 | 2 | 2 | 1 | 2 | 0 | 2 | 0 | 11 | 1 |  |  |  | 1 |  |  |  |  | 0 | 1 |
| **18** | 52 | 3 | 65 | a | 1 | 2 | 1 | 4 | 2 | 0 | 0 | 1 | 2 | 2 | 0 | 0 | 7 | 1 |  |  |  | 1 | 1 |  |  |  | 1 | 2 |
| **19** | 6 | 1 | 27 | a | 3 | 2 | 1 | 6 | 2 | 2 | 0 | 2 | 2 | 2 | 2 | 1 | 13 | 1 |  |  |  | 1 | 1 |  | 1 |  | 2 | 3 |
| **20** | 20 | 2 | 40 | a | 3 | 1 | 0 | 4 | 2 | 2 | 0 | 2 | 2 | 0 | 0 | 0 | 8 | 1 |  |  |  | 1 |  |  |  |  | 0 | 1 |
| **21** | 8 | 3 | 23 | a | 1 | 5 | 2 | 8 | 2 | 2 | 0 | 2 | 2 | 2 | 0 | 0 | 10 | 1 |  | 1 |  | 2 |  |  |  |  | 0 | 2 |
| **22** | 15 | 4 | 37 | a | 2 | 3 | 2 | 7 | 0 | 2 | 1 | 0 | 2 | 2 | 2 | 0 | 11 | 1 |  | 1 |  | 2 |  | 1 |  |  | 1 | 3 |
| **23** | 18 | 6 | 46 | a | 3 | 0 | 0 | 3 | 2 | 0 | 2 | 2 | 2 | 0 | 1 | 0 | 7 | 0 |  |  |  | 0 |  |  |  |  | 0 | 0 |
| **24** | 12 | 3 | 31 | a | 0 | 0 | 1 | 1 | 2 | 2 | 2 | 2 | 2 | 0 | 0 | 0 | 10 | 1 |  | 1 |  | 2 | 1 |  | 1 |  | 2 | 4 |
| **25** | 20 | 4 | 37 | a | 4 | 6 | 3 | 13 | 2 | 2 | 0 | 0 | 2 | 0 | 2 | 1 | 9 | 1 |  | 1 |  | 2 |  |  |  | 1 | 1 | 3 |
| **26** | 30 | 3 | 44 | a | 0 | 0 | 0 | 0 | 0 | 2 | 2 | 0 | 2 | 0 | 0 | 0 | 8 | 1 |  |  |  | 1 |  |  |  |  | 0 | 1 |
| **27** | 48 | 1 | 60 | a | 2 | 3 | 0 | 5 | 2 | 2 | 2 | 2 | 2 | 2 | 0 | 1 | 13 | 1 |  |  |  | 1 |  |  |  |  | 0 | 1 |
| **28** | 30 | 1 | 46 | a | 4 | 0 | 0 | 4 | 2 | 2 | 2 | 0 | 2 | 0 | 2 | 0 | 10 | 1 | 1 |  |  | 2 |  |  |  |  | 0 | 2 |
| **29** | 40 | 2 | 0 | a | 0 | 6 | 1 | 7 | 2 | 0 | 0 | 0 | 0 | 2 | 2 | 0 | 4 | 0 |  |  |  | 0 | 1 |  |  |  | 1 | 1 |
| **30** | 0 | 1 | 35 | a | 1 | 0 | 0 | 1 | 0 | 2 | 2 | 0 | 2 | 2 | 2 | 2 | 14 | 1 |  |  |  | 1 |  |  |  |  | 0 | 1 |
| **31** | 3 | 2 | 19 | a | 1 | 0 | 0 | 1 | 2 | 2 | 2 | 2 | 2 | 0 | 2 | 1 | 13 | 1 |  |  |  | 1 |  |  |  |  | 0 | 1 |
| **32** | 20 | 8 | 28 | a | 3 | 2 | 1 | 6 | 2 | 2 | 2 | 2 | 2 | 1 | 2 | 0 | 13 | 1 |  | 1 |  | 2 |  |  |  |  | 0 | 2 |
| **33** | 2 | 1 | 26 | a | 4 | 6 | 1 | 11 | 2 | 2 | 2 | 0 | 2 | 0 | 2 | 0 | 10 | 1 |  |  |  | 1 |  |  |  | 1 | 1 | 2 |
| **34** | 24 | 1 | 38 | a | 1 | 0 | 2 | 3 | 2 | 2 | 2 | 2 | 2 | 2 | 2 | 0 | 14 | 1 |  |  |  | 1 |  |  |  | 1 | 1 | 2 |
| **35** | 43 | 2 | 55 | a | 1 | 0 | 2 | 3 | 0 | 2 | 2 | 2 | 2 | 0 | 2 | 0 | 12 | 0 | 1 | 1 |  | 2 |  |  |  | 1 | 1 | 3 |
| **36** | 12 | 4 | 24 | a | 0 | 0 | 0 | 0 | 2 | 2 | 0 | 0 | 0 | 2 | 0 | 0 | 6 | 1 |  | 1 |  | 2 | 1 |  |  |  | 1 | 3 |
| **37** | 51 | 2 | 60 | a | 0 | 0 | 6 | 6 | 0 | 2 | 2 | 2 | 2 | 2 | 2 | 0 | 14 | 1 | 1 |  |  | 2 | 1 |  |  | 1 | 2 | 4 |
| **38** | 29 | 3 | 39 | a | 6 | 6 | 5 | 17 | 2 | 0 | 0 | 0 | 0 | 0 | 2 | 2 | 4 | 1 |  |  | 1 | 2 |  |  |  | 1 | 1 | 3 |
| **39** | 35 | 1 | 40 | a | 1 | 5 | 2 | 8 | 2 | 2 | 0 | 0 | 0 | 0 | 0 | 0 | 4 | 1 | 1 | 1 |  | 3 | 1 |  |  |  | 1 | 4 |
| **40** | 33 | 4 | 48 | a | 0 | 0 | 0 | 0 | 0 | 2 | 2 | 2 | 2 | 0 | 2 | 0 | 12 | 1 |  |  |  | 1 |  |  |  |  | 0 | 1 |
| **41** | 25 | 4 | 48 | c | 0 | 3 | 0 | 3 | 1 | 2 | 2 | 2 | 2 | 0 | 1 | 1 | 12 | 1 |  | 1 |  | 2 |  |  | 1 |  | 1 | 3 |
| **42** | 18 | 2 | 32 | c | 2 | 5 | 0 | 7 | 2 | 2 | 2 | 2 | 2 | 0 | 1 | 1 | 12 | 1 |  |  |  | 1 |  |  |  |  | 0 | 1 |
| **43** | 12 | 5 | 40 | c | 2 | 3 | 1 | 6 | 2 | 2 | 1 | 2 | 2 | 1 | 1 | 2 | 13 |  |  |  |  | 0 |  |  |  |  | 0 | 0 |
| **44** | 17 | 3 | 32 | c | 3 | 0 | 4 | 7 | 0 | 1 | 1 | 1 | 1 | 1 | 1 | 1 | 8 | 1 |  |  |  | 1 | 1 |  |  |  | 1 | 2 |
| **45** | 28 | 1 | 45 | c | 2 | 0 | 1 | 3 | 2 | 2 | 2 | 1 | 2 | 1 | 2 | 0 | 12 | 1 | 1 |  |  | 2 |  |  |  |  | 0 | 2 |
| **46** | 45 | 5 | 55 | c | 2 | 6 | 1 | 9 | 2 | 2 | 2 | 1 | 2 | 1 | 2 | 0 | 12 | 1 |  |  |  | 1 |  | 1 | 1 | 1 | 3 | 4 |
| **47** | 8 | 1 | 38 | c | 3 | 0 | 3 | 6 | 2 | 2 | 0 | 1 | 2 | 2 | 2 | 0 | 11 | 1 |  |  |  | 1 | 1 |  |  | 1 | 2 | 3 |
| **48** | 2 | 1 | 43 | c | 0 | 0 | 0 | 0 | 0 | 2 | 1 | 2 | 2 | 1 | 2 | 1 | 12 | 0 |  |  |  | 0 |  |  |  |  | 0 | 0 |
| **49** | 25 | 5 | 37 | c | 4 | 4 | 1 | 9 | 1 | 0 | 0 | 0 | 0 | 0 | 0 | 0 | 0 | 1 | 1 |  |  | 2 |  |  |  |  | 0 | 2 |
| **50** | 20 | 3 | 41 | c | 2 | 3 | 1 | 6 | 2 | 2 | 2 | 2 | 2 | 2 | 2 | 1 | 15 | 1 | 1 | 1 |  | 3 |  |  |  |  | 0 | 3 |
| **51** | 12 | 3 | 24 | c | 3 | 6 | 1 | 10 | 0 | 2 | 2 | 2 | 2 | 2 | 2 | 1 | 15 | 1 |  |  |  | 1 |  |  |  |  | 0 | 1 |
| **52** | 20 | 6 | 31 | c | 3 | 6 | 1 | 10 | 2 | 1 | 2 | 2 | 2 | 0 | 2 | 1 | 12 | 1 | 1 |  |  | 2 | 1 |  | 1 |  | 2 | 4 |
| **53** | 19 | 4 | 31 | c | 2 | 6 | 3 | 11 | 2 | 2 | 1 | 1 | 2 | 1 | 2 | 1 | 11 | 1 |  |  |  | 1 |  |  | 1 |  | 1 | 2 |
| **54** | 26 | 2 | 42 | c | 3 | 5 | 0 | 8 | 0 | 2 | 1 | 2 | 2 | 2 | 1 | 1 | 13 | 0 |  |  |  | 0 |  |  |  |  | 0 | 0 |
| **55** | 50 | 3 | 62 | c | 2 | 0 | 1 | 3 | 2 | 0 | 0 | 0 | 0 | 0 | 0 | 0 | 0 | 1 |  |  |  | 1 |  |  |  | 1 | 1 | 2 |
| **56** | 30 | 2 | 62 | c | 4 | 6 | 1 | 11 | 2 | 2 | 1 | 1 | 1 | 1 | 1 | 1 | 10 | 1 |  |  |  | 1 | 1 |  |  | 1 | 2 | 3 |
| **57** | 0.17 | 1 | 51 | c | 4 | 4 | 5 | 13 | 2 | 2 | 1 | 1 | 1 | 0 | 1 | 1 | 9 | 1 |  |  |  | 1 | 1 |  |  | 1 | 2 | 3 |
| **58** | 20 | 2 | 46 | c | 3 | 4 | 2 | 9 | 2 | 2 | 1 | 1 | 1 | 1 | 1 | 1 | 10 | 1 |  |  |  | 1 | 1 |  |  | 1 | 2 | 3 |
| **59** | 12 | 4 | 33 | c | 1 | 6 | 5 | 12 | 2 | 2 | 2 | 1 | 2 | 2 | 2 | 1 | 13 | 1 |  | 1 |  | 2 | 1 |  |  | 1 | 2 | 4 |
| **60** | 38 | 3 | 49 | c | 3 | 3 | 0 | 6 | 2 | 1 | 2 | 1 | 2 | 1 | 2 | 1 | 12 | 1 |  | 1 |  | 2 |  |  |  |  | 0 | 2 |
| **61** | 18 | 4 | 41 | c | 1 | 5 | 1 | 7 | 2 | 2 | 0 | 0 | 1 | 1 | 2 | 1 | 9 | 0 | 1 | 1 |  | 2 |  |  |  | 1 | 1 | 3 |
| **62** | 32 | 1 | 40 | c | 0 | 0 | 2 | 2 | 0 | 0 | 0 | 0 | 0 | 0 | 0 | 0 | 0 | 0 |  | 1 |  | 1 |  |  |  | 1 | 1 | 2 |
| **63** | 30 | 4 | 42 | c | 3 | 0 | 0 | 3 | 1 | 0 | 0 | 0 | 0 | 0 | 0 | 0 | 0 | 1 |  | 1 |  | 2 |  |  |  |  | 0 | 2 |
| **64** | 16 | 8 | 39 | c | 0 | 0 | 0 | 0 | 2 | 2 | 1 | 0 | 1 | 1 | 1 | 0 | 8 | 1 |  | 1 |  | 2 |  |  |  |  | 0 | 2 |
| **65** | 32 | 2 | 44 | c | 2 | 3 | 4 | 9 | 2 | 2 | 0 | 2 | 2 | 1 | 0 | 0 | 9 | 1 |  | 1 |  | 2 | 1 |  |  | 1 | 2 | 4 |
| **66** | 34 | 2 | 46 | c | 2 | 5 | 4 | 11 | 2 | 2 | 2 | 2 | 2 | 0 | 2 | 0 | 12 | 1 | 1 |  |  | 2 | 1 |  | 1 | 1 | 3 | 5 |
| **67** | 6 | 3 | 16 | c | 3 | 8 | 1 | 12 | 2 | 2 | 2 | 1 | 2 | 0 | 2 | 0 | 11 | 1 |  | 1 |  | 2 |  |  |  |  | 0 | 2 |
| **68** | 21 | 4 | 33 | c | 4 | 6 | 1 | 11 | 2 | 2 | 0 | 1 | 0 | 2 | 0 | 0 | 7 | 1 | 1 |  | 1 | 3 |  |  |  |  | 0 | 3 |
| **69** | 50 | 1 | 62 | c | 2 | 0 | 4 | 6 | 2 | 0 | 0 | 2 | 2 | 0 | 1 | 3 | 8 | 1 |  | 1 |  | 2 | 1 |  | 1 |  | 2 | 4 |
| **70** | 7 | 3 | 19 | c | 3 | 8 | 1 | 12 | 1 | 2 | 0 | 1 | 2 | 0 | 0 | 0 | 7 | 1 |  | 1 |  | 2 |  |  |  | 1 | 1 | 3 |
| **71** | 38 | 6 | 50 | c | 3 | 0 | 2 | 5 | 2 | 2 | 0 | 0 | 2 | 2 | 2 | 1 | 11 | 1 | 1 |  |  | 2 | 1 |  | 1 |  | 2 | 4 |
| **72** | 20 | 1 | 31 | c | 1 | 3 | 0 | 4 | 2 | 2 | 2 | 0 | 2 | 2 | 1 | 0 | 11 | 1 |  |  |  | 1 | 1 |  |  |  | 1 | 2 |
| **73** | 11 | 2 | 38 | c | 0 | 0 | 0 | 0 | 2 | 0 | 0 | 0 | 0 | 0 | 0 | 2 | 2 | 1 |  | 1 |  | 2 |  |  |  |  | 0 | 2 |
| **74** | 28 | 1 | 40 | c | 2 | 3 | 0 | 5 | 2 | 2 | 0 | 0 | 2 | 2 | 2 | 0 | 10 | 1 |  |  |  | 1 |  |  |  |  | 0 | 1 |
| **75** | 30 | 1 | 44 | c | 1 | 3 | 2 | 6 | 2 | 2 | 0 | 0 | 2 | 0 | 1 | 0 | 7 | 1 |  | 1 | 1 | 3 |  |  |  |  | 0 | 3 |
| **76** | 25 | 7 | 48 | c | 3 | 2 | 3 | 8 | 2 | 0 | 0 | 0 | 2 | 0 | 2 | 0 | 6 | 1 |  | 1 |  | 2 | 1 |  | 1 |  | 2 | 4 |
| **77** | 40 | 1 | 70 | c | 1 | 0 | 2 | 3 | 2 | 2 | 2 | 0 | 2 | 0 | 2 | 0 | 10 | 1 |  | 1 |  | 2 |  |  |  |  | 0 | 2 |
| **78** | 40 | 2 | 49 | c | 2 | 1 | 1 | 4 | 2 | 2 | 2 | 0 | 2 | 2 | 0 | 1 | 11 | 1 | 1 | 1 | 1 | 4 | 1 |  |  |  | 1 | 5 |
| **79** | 40 | 3 | 52 | c | 1 | 5 | 1 | 7 | 2 | 0 | 0 | 0 | 0 | 0 | 0 | 0 | 2 | 1 | 1 | 1 | 1 | 4 |  |  |  |  | 0 | 4 |
| **80** | 6 | 5 | 28 | c | 2 | 0 | 1 | 3 | 2 | 0 | 0 | 0 | 0 | 0 | 0 | 0 | 0 |  |  | 1 |  | 1 | 1 |  |  |  | 1 | 2 |
| **81** | 37 | 2 | 37 | c | 2 | 0 | 1 | 3 | 2 | 0 | 0 | 0 | 0 | 0 | 0 | 0 | 0 | 1 |  |  | 1 | 2 |  |  |  | 1 | 1 | 3 |
| **82** | 3 | 5 | 31 | c | 5 | 6 | 3 | 14 | 2 | 2 | 2 | 2 | 2 | 2 | 2 | 0 | 14 | 1 |  |  |  | 1 | 1 |  |  | 1 | 2 | 3 |
| **83** | 12 | 4 | 33 | c | 2 | 1 | 0 | 3 | 2 | 0 | 0 | 0 | 0 | 2 | 2 | 1 | 5 | 1 |  |  |  | 1 | 1 |  | 1 |  | 2 | 3 |
| **84** | 25 | 3 | 42 | c | 0 | 0 | 0 | 0 | 2 | 0 | 1 | 0 | 2 | 2 | 2 | 0 | 7 | 1 | 1 |  |  | 2 |  |  |  |  | 0 | 2 |
| **85** | 40 | 4 | 52 | c | 3 | 6 | 2 | 11 | 2 | 2 | 2 | 2 | 2 | 0 | 0 | 0 | 10 | 1 |  | 1 |  | 2 | 1 |  |  |  | 1 | 3 |
| **86** | 35 | 2 | 47 | c | 2 | 0 | 1 | 3 | 2 | 2 | 2 | 2 | 2 | 0 | 2 | 0 | 12 | 1 |  | 1 |  | 2 |  |  |  |  | 0 | 2 |
| **87** | 51 | 1 | 63 | c | 0 | 0 | 1 | 1 | 2 | 0 | 2 | 0 | 2 | 2 | 1 | 0 | 9 | 0 |  |  |  | 0 |  |  |  |  | 0 | 0 |
| **88** | 0 | 7 | 0 | c | 4 | 3 | 3 | 10 | 2 | 2 | 2 | 2 | 2 | 2 | 2 | 0 | 14 | 1 |  |  |  | 1 | 1 |  | 1 |  | 2 | 3 |
| **89** | 35 | 2 | 47 | c | 2 | 0 | 1 | 3 | 2 | 2 | 2 | 2 | 2 | 2 | 2 | 1 | 13 | 1 |  |  | 1 | 2 |  |  |  | 1 | 1 | 3 |
| **90** | 20 | 2 | 63 | c | 2 | 0 | 0 | 2 | 0 | 0 | 0 | 0 | 0 | 0 | 0 | 0 | 0 | 1 |  |  |  | 1 |  |  |  |  | 0 | 1 |
| **91** | 25 | 2 | 43 | c | 5 | 3 | 1 | 9 | 2 | 2 | 2 | 2 | 2 | 2 | 0 | 1 | 13 | 1 | 1 | 1 |  | 3 |  |  |  | 1 | 1 | 4 |
| **92** | 38 | 6 | 50 | C | 1 | 1 | 0 | 2 | 2 | 2 | 0 | 0 | 0 | 0 | 0 | 0 | 4 | 1 |  |  |  | 1 |  |  |  |  | 0 | 1 |

**The R code used for creating data in Table 5 and 6.**

getwd()

setwd("D:/working/R studio/master/analysis R")

data1 <- read.csv(file.choose(), header=T)

summary(data1)

shapiro.test(data1$k.sum)

shapiro.test(data1$a.sum)

shapiro.test(data1$p.sum)

shapiro.test(data1$experiance)

shapiro.test(data1$age)

#correlation

cortt<-cor.test(data1$k.sum,data1$a.sum,method="spearman")

cortt

cortt2<-cor.test(data1$k.sum,data1$p.sum,method="spearman")

cortt2

cortt3<-cor.test(data1$a.sum,data1$p.sum,method="spearman")

cortt3

#cor.test KAP vs experience

corex_p<-cor.test(data1$experiance,data1$p.sum,method="spearman")

corex_p

corex_k<-cor.test(data1$experiance,data1$k.sum,method="spearman")

corex_k

corex_a<-cor.test(data1$experiance,data1$a.sum,method="spearman")

corex_a

#cor.test KAP vs age

corage_k<-cor.test(data1$age,data1$k.sum,method="spearman")

corage_k

corage_p<-cor.test(data1$age,data1$p.sum,method="spearman")

corage_p

corage_a<-cor.test(data1$age,data1$a.sum,method="spearman")

corage_a

sum<-summary(lm(data1$k.sum~data1$p.sum))

sum

**Raw data used for creating a boxplot that showed in figure 3. The boxplot was created using the R program (version 4.1.0).**

| **Number**  **of**  **mahout** | **Age class of**  **elephant under care**  (c = calve)  (a = adult) | **Summary**  **of**  **knowledge**  **score** | **Summary**  **of**  **attitude**  **score** | **Summary**  **of**  **practice**  **score** |
| --- | --- | --- | --- | --- |
| 1 | c | 3 | 12 | 3 |
| 2 | c | 7 | 12 | 1 |
| 3 | a | 1 | 13 | 1 |
| 4 | c | 6 | 13 | 0 |
| 5 | a | 0 | 12 | 2 |
| 6 | c | 7 | 8 | 2 |
| 7 | a | 13 | 11 | 2 |
| 8 | c | 3 | 12 | 2 |
| 9 | c | 9 | 12 | 4 |
| 10 | c | 6 | 11 | 3 |
| 11 | a | 2 | 14 | 3 |
| 12 | a | 7 | 15 | 1 |
| 13 | c | 0 | 12 | 0 |
| 14 | c | 9 | 0 | 2 |
| 15 | c | 6 | 15 | 3 |
| 16 | c | 10 | 15 | 1 |
| 17 | a | 4 | 0 | 3 |
| 18 | a | 4 | 12 | 1 |
| 19 | c | 10 | 12 | 4 |
| 20 | c | 11 | 11 | 2 |
| 21 | c | 8 | 13 | 0 |
| 22 | a | 4 | 11 | 2 |
| 23 | c | 3 | 0 | 2 |
| 24 | c | 11 | 10 | 3 |
| 25 | c | 13 | 9 | 3 |
| 26 | c | 9 | 10 | 3 |
| 27 | a | 3 | 16 | 2 |
| 28 | c | 12 | 13 | 4 |
| 29 | c | 6 | 12 | 2 |
| 30 | a | 2 | 17 | 4 |
| 31 | a | 1 | 15 | 2 |
| 32 | a | 3 | 11 | 0 |
| 33 | c | 7 | 9 | 3 |
| 34 | a | 2 | 12 | 2 |
| 35 | a | 10 | 13 | 2 |
| 36 | a | 9 | 14 | 2 |
| 37 | c | 2 | 0 | 2 |
| 38 | c | 3 | 0 | 2 |
| 39 | c | 0 | 8 | 2 |
| 40 | a | 5 | 13 | 2 |
| 41 | a | 6 | 11 | 1 |
| 42 | a | 4 | 7 | 2 |
| 43 | a | 6 | 13 | 3 |
| 44 | a | 4 | 8 | 1 |
| 45 | c | 9 | 9 | 4 |
| 46 | c | 11 | 12 | 5 |
| 47 | c | 12 | 11 | 2 |
| 48 | a | 8 | 10 | 2 |
| 49 | a | 7 | 11 | 3 |
| 50 | c | 11 | 7 | 3 |
| 51 | c | 6 | 8 | 4 |
| 52 | a | 3 | 7 | 0 |
| 53 | c | 12 | 7 | 3 |
| 54 | c | 5 | 11 | 4 |
| 55 | c | 4 | 11 | 2 |
| 56 | a | 1 | 10 | 4 |
| 57 | c | 0 | 2 | 2 |
| 58 | a | 13 | 9 | 3 |
| 59 | c | 5 | 10 | 1 |
| 60 | c | 6 | 7 | 3 |
| 61 | c | 8 | 6 | 4 |
| 62 | c | 3 | 10 | 2 |
| 63 | a | 0 | 8 | 1 |
| 64 | c | 4 | 11 | 5 |
| 65 | a | 5 | 13 | 1 |
| 66 | c | 7 | 2 | 4 |
| 67 | a | 4 | 10 | 2 |
| 68 | a | 7 | 4 | 1 |
| 69 | a | 1 | 14 | 1 |
| 70 | c | 3 | 0 | 2 |
| 71 | c | 3 | 0 | 3 |
| 72 | c | 14 | 14 | 3 |
| 73 | a | 1 | 13 | 1 |
| 74 | a | 6 | 13 | 2 |
| 75 | c | 3 | 5 | 3 |
| 76 | c | 0 | 7 | 2 |
| 77 | c | 11 | 10 | 3 |
| 78 | c | 3 | 12 | 2 |
| 79 | c | 1 | 9 | 0 |
| 80 | a | 11 | 10 | 2 |
| 81 | a | 3 | 14 | 2 |
| 82 | a | 3 | 12 | 3 |
| 83 | a | 0 | 6 | 3 |
| 84 | c | 10 | 14 | 3 |
| 85 | a | 6 | 14 | 4 |
| 86 | c | 3 | 13 | 3 |
| 87 | c | 2 | 0 | 1 |
| 88 | a | 17 | 4 | 3 |
| 89 | a | 8 | 4 | 4 |
| 90 | c | 9 | 13 | 4 |
| 91 | a | 0 | 12 | 1 |
| 92 | C | 2 | 4 | 1 |

**The R code used for creating boxplot that showed in Figure 3.**

getwd ()

setwd (“D:/working/R studio/master/analysis R”)

ca.graph <- read.csv (file.choose ( ) , header = T)

attach (ca.graph)

library (tidyverse)

# it has ggplot2 package

library (cowplot)

# it allows you to save figures in .png file

Library (smplot2)

install.packages (“ggplot2”)

library (“ggplot2”)

library (“ggpubr”)

library (reshape2)

multi <- melt (ca.graph, id = “cal_adu”)

head (multi)

ggplot (multi, aes (x = variable, y = value, color = cal_adu)) +

# ggplot function

Geom_bloxplot ()
