# Supplementary material for: Unlocking insights: Mahout’s perceptions and practices in managing Elephant Endotheliotropic Herpesvirus (EEHV) infection among captive Asian elephants in Surin province, Thailand
Source: PLoS One. 2024 Nov 12;19(11):e0295869. doi: 10.1371/journal.pone.0295869 (PMC11556736; doi:10.1371/journal.pone.0295869)
Supplement: Attachment — Submitted filename: Response to Reviewers.docx [file pone.0295869.s002.docx]

Faculty of Veterinary Medicine

Kasetsart University, Kamphaeng Saen

Nakhon Pathom 73140 Thailand

To:	The Editor-in-Chief of PLOS ONE

Date:	20 June 2024

Subject: Submission of revised manuscript

Dear Editor,

I am re-submitting the manuscript PONE-D-23-38946 “*Unlocking Insights: Mahout’s Perceptions and Practices in Managing Elephant Endotheliotropic Herpesvirus (EEHV) infection Among Captive Asian Elephants in Surin province, Thailand*”, for further review. Comments from the editor and reviewers have been addressed and substantial changes were made. Details of responses regarding to the reviewer’s comment are as follows;

**Journal requirements:**

1. Please ensure that your manuscript meets PLOS ONE’s style requirements.

- The authors had checked throughout the manuscript the ensure that it meets PLOS ONE’s style requirements.

2. Please state what role of the funders took in this study.

- The funders had no role in study design, data collection and analysis, decision to publish, or preparation of the manuscript.

3. Please remove any funding-related text from the manuscript.

- The funding-related text had been removed from the acknowledgement in the manuscript.

4. The copyright issue of Figure 1.

- Figure 1 had been removed from the manuscript.

**Reviewer1:**

*General comments*

Reviewer 1 has recommended a complete reframing of the paper by comparing different stakeholders/areas or adding more data collection and reanalyzing the data.

- *Response in Page 4, line 84-85, Page 23, line 446-448*: due to the limited of funding and study timeframe, the authors were unable to compare the results with other stakeholders/areas, however, Surin province is the place where most of the baby elephants were born and been transported to other area of Thailand. Therefore, it counts as a biggest population of captive elephant in Thailand with unique traditional management system. Beginning the study in Surin province could provide a database for captive elephant population in Thailand. In addition, the authors have added information on the history of EEHV diagnosed/suspected cases in Surin and also reanalyze some data, as reviewer suggested (see details below).

*Specific comments and editorial suggestions*

- Title: suggest removing “Unlocking insights:”, not clear which insights are referred to, and whether they are unlocked by the survey?
  - *Response in Title* *(page 1):* the authors would like to remain “Unlocking insight”, if possible. As it refers to the in-depth personal data collection from elephant keepers which has not been extensively explored before in Thailand. However, if the editorial team agreed on removing ‘unlocking insights’, the authors are also fine to remove.
- Abstract: Line 32: unclear what “disease information” means
- *Response in Page 2, line 33-34*: change to “nature of disease and preventive measures” to clarify the “disease information”.
- Abstract: Line 37: do you mean “contract EEHV”?
- Response in *Page 2, line 39*: changed to “receive”.

-	L70: change sentence “… medicine, the threat of elephant endotheliotropic…was recently prioritized. EEHV is a fatal hemorrhagic … documented in Thailand ….”

- Response in *Page 4, line 81-83*: changed as reviewer suggested.

- L73: consider changing “carries” into “induces/is responsible for”

- Response in *Page 5, line 86*: changed to “is responsible for”.

- L78: add “years” after “1 and 8”

- Response in *Page 5, line 93*: added “years” as reviewer suggested.

- L82: change “promising” into “conclusive” or efficient

- Response in *Page 5, line 97*: changed to “conclusive”.

- L93: repeat “specific”

- Response in *Page 6, line 109*: changed to “key”.

- L97: do you mean “extent” instead of “impact”

- Response in *Page 6, line 114*: changed to “extent”.

- L107: change “the survey” to “This survey” or “Our survey”

- Response in *Page 6, line 126*: changed to “This survey”.

- L110: add “activities” after “… elephant tourism activities”

- Response in *Page 6, line 129*: added “activities” as reviewer suggested.

- L122: correct “… area presented …”

- Figure 1 (the map) was removed.

- L124/Figure legend: correct “Map of” instead of “Mapping”

- Figure 1 (the map) was removed.

- L125/Figure legend: correct “sampled” instead of “sampling”

- Figure 1 (the map) was removed.

- L126/Figure legend: remove “The display .. QGIS [22]” from the figure legend and possibly add to Mat&Meth

- Figure 1 (the map) was removed.

- L131: correct “semi-structured”

- Response in *Page 8, line 149*: changed to “semi-structured”.

- L131: clarify “observations” of what?

- Response in *Page 8, line 149*: changed “observation method” to “field observation.”
- Response in *Page 9, line 172-174:* added the definition of field observation “In addition, the field observation was applied to assess husbandry, waste management, and general care provided to elephants, in comparison to insights gathered through interviews”.

- L133: give n = mahouts with elephant < 10 years

- Response in *Page 8, line 152:* added the number of mahouts (n=41).

- L153: and whole section Mat&Metho: clarify why the number of “points”/give different weights is different between subjects?

- Response in Section “Data collection method” (*Page 8-10)*: rewritten and added relevant information as reviewer suggested.

- L153: suggestion to add a sensitivity analysis clarifying the impact of the subjective weighting/subject on the end-results and conclusions

- Response in section “Assessing the KAP scores in relation to mahout backgrounds” (Page 19-22): In this study, sensitivity analysis was conducted using correlation analysis test and Wilcoxon, and the **Mann-Whitney U Test**

- L182/Statistical analysis: remove unnecessary reference to Excel

- Response in *Page 11, line 219*: removed Microsoft Excel

- L186: clarify which stat test were used to compared which variables/group

- Response in *Page 11, line 221-228*: added “The statistical analysis included correlation testing to examine the relationships between knowledge, attitude, and practice scores, and the mahouts' demographics (such as age and experience in elephant handling). Additionally, the Wilcoxon test was used to compare KAP scores between mahouts who care for elephant calves and those who care for adult elephants. While the Mann-Whitney U test was used to compare KAP scores between the experienced in EEHV infected elephant groups and the non-experience groups.”

- L190: clarify how many mahouts in total are involved in EKP

- Response in *Page 11, line 232-233*: added “From the total of 200 mahouts in EKP, this study included 92 participants (mahouts) from 81 households”.

- L196: unclear by whom the system is known, and maybe indicate the Thai name of the system (if it’s a translation)?

- Response in *Page 12, line 237-240*: removed one-by-one system to prevent confusion and added data regarding to the elephant management in this study area.

- Fig2, Fig3, Fig4: remove the pictures, which are not very useful, especially 3 and 4

- Response in *Page 12, line 249-252*: the authors would like to remain the pictures as they were illustrated the elephant husbandry in this study areas, which probably useful for the reader. However, the authors combine Fig 3 and 4 into single picture to present the resting area of elephant in this study.

- L211-2: Clarify semantics: which language was used, which term in vernacular language used…

- Response in *Page 14, line 262-270*: rewritten and added more data for clarification.

- L211: What question was asked to the mahouts exactly to assess if the respondents knew about EEHV?

- Response in *Page 14, line 261-262*: The interview was begun by directly asking "Do you know herpesvirus in elephants?".

- L217/Table 2 title: replace “according” by “regarding”?

- Response in *Page 14 (Table 2)*: changed to “regarding”.

- L217/Table 2: Why no question was asked about potential treatment of EEHV? Besides current veterinary knowledge, maybe some respondents may have some ideas/experiences of treatment (e.g. symptomatic treatment?)

- The participants were asked about potential treatment of EEHV and their experience in EEHV treatment, however, the data was not showed in the original manuscript. The data regarding to EEHV treatment was added in the revision (see *Page 23, line 436-443*): “Moreover, interviews conducted with three mahouts whose elephants experienced clinical EEHV infections in 2017 revealed their belief in both traditional methods and modern medication. The elephants were treated at home because the mahouts believed that transporting the animals to the hospital would cause undue stress. However. antiviral drugs were given alongside spiritual practices performed by a local hermit. These practices included praying, drinking holy water, and spraying consecrated white liquor”.

- L219: change “the study” into “our study”

- Response in *Page 15, line 278*: changed to “this study” for consistency throughout the document.

- L225: what was exactly the question asked regarding the transmission route? Did you leave the question open for possible (local knowledge) alternative etiologies?

- Yes, the question was open questions about how EEHV was transmitted? The final question was a close question to confirm the knowledge of main transmission route, the final question was “EEHV can be transmitted from elephant to elephant, yes or no?”. Moreover, additional data was added to clarify the knowledge on transmission (see *Page 15, line 287-293*).

- L238: consider replacing “concerning” by “associated with newly”

- Response in *Page 16, line 310*: changed to “associated with newly”.

- Table 3: correct “1. Calve elephant” and revise unclear “5. Infection EEHV from infected?

- Response in Table 3 *(Page 17):* the misspelling was corrected, and the attitude subject was rewritten to make it clearer.

- L244: clarify what are “the concerns”

- Response in *Page 16, line 314*: added “which could interfere protective immune development”.

- L247-8: revise: an elephant can’t “roam freely” if it is chained

- Response in *Page 16, line 318*: rewritten to “freely browse for food in the forested area with a long chain”.

- L247: revise “under natural elements”

- Response in *Page 17, line 318*: removed “under natural elements” to prevent the confusion.

- L265: provide “evidence”: what data collect providing evidence?

- Response in *Page 18, line 341-342*: added “the EKP elephant movement logbook”.

- L268: clarify how Covid-19 restrictions/lock-downs affected elephant movements and what evidence there is that it altered the contact patterns between elephants, and EEHV spread?

- Response in *Page 18-19, line 347-359*: added the effect of COVID-19 on elephant movement.

- L275: clarify “... the cleaning process needed to be specified”.

- Response in *Page 19, line 365-366*: rewritten and changed to “no cleaning process was performed”.

- L280: revise title “the relation…”

- Response in *Page 19, line 370*: changed the subheading to “Assessing the KAP score in relation to mahout backgrounds”.

- L281-4: unclear 1^st^ sentence: remove?

- Response in *Page 19, line 371*: removed and rewritten.

- L291: revise title “correlation between KAP scores”

- Response in *Page 20, line 383 (Table 5)*: changed to “Comparative analysis of KAP scores with age of mahout and elephant rearing experience”.

- L303-4: and throughout the next: specify “knowledge and practices related to EEHV”

- Response in *Page 21, line 402*: added “related to EEHV”.

- L313: revise “knowledge scores of mahouts caring for adult elephants and those caring for young elephants

- Response in *Figure 3 title (Page 21):* changed Fig 5 to Fig 3 and changed title to “The boxplot compared KAP scores of mahouts caring for adult elephants and those caring for young elephants”.

- L323: add reference or anthropological and historical records reporting how and by whom “traditional practices” handed down through generations”

- Response in *Page 22, line 428*: added the reference.

- L325: provide data supporting the allegation that cultural and religious beliefs influence…” no evidence provided with the KAP data presented

- Response in *Page 22-23, line 426 – 449:* added supporting data as reviewer suggested.

- L340: give precise example of “other illnesses” and differential diagnostic?

- Response in *Page 24, line 466 - 469*: added “For example, swelling of face and neck in elephants was sometimes mistaken to hemorrhagic septicemia, allergies, or obesity. This could easily be differentiated from EEHV through PCR or hematology tests. However, this misconception often led to delays in notifying veterinarians.”.

- L343: clarify what is “KAP model”

- Response in *Page 24, line 472 - 474*: added “KAP model, which represents the interconnection among knowledge, attitudes, and practices that can lead to changes in individual behaviors”.

- L343-4: this sentence is puzzling .. why did you calculate the correlation coefficients if they are not necessarily correlated?

- Response in *Page 24-25, line 474- 478*: added “This model demonstrated that knowledge and attitude influence practice, even if they are not necessarily correlated. Additionally, improved understanding of EEHV among mahouts can lead to better practices for disease prevention, which may not have been statistically correlated previously.”

- L345: clarify “nature of herpesvirus”

- Response in *Page 26, 497 - 499*: added “The nature of herpesvirus, which could establish latency and subsequently reactivate, in humans and other animal species is directly related to stress levels and immune response”.

- L353: indicated which “other infection” exactly

- Response in *Page 26, line 507 - 508*: added “such as tuberculosis into the project area”.

- L372: add “risk of infection by EEHV”

- Response in *Page 28, line 542*: added “risk of infection by EEHV”.

- L375: clarify where and when “elephant freely explore..” and give evidence/quote mahouts saying “herbs and trees support their recovery (from what/how)?

- Response in *Page 22-23, line 426-436*: added more information on traditional belief of mahouts in the study area.

- L377-8: clarify why they would be marginalized from their community/other mahouts?

- Response in *Page 28, line 551-554*: added “In many traditional communities, long-established cultural practices are highly valued and strictly followed, which quite challenge to bring new information or modern medicine to the community.”

- L393: provide indications how you intend to achieve this challenging goal; “integrate newly found knowledge…”

- Response in *Page 29, line 560-565*: added data as reviewer suggested.

- Bibliography: the format was not consistent throughout

- Response in *Bibliography (Page 30-38)*: corrected the format of bibliography.

- Map: unclear

- Removed the map (Figure 1)

- Pictures: remove?

- Remain the figures as they represented the management in the study areas but combined them into one figure.

**Reviewer2:**

1. Age and KAP: The current age of respondent range of 15-60 years is quite broad. It would be beneficial to recategorize age groups for a more detailed comparison.

- Response in *Table 1 (Page 13):* recategorized the age group of respondents to five groups, including ≤ 15, 16-30, 31-45, 46-60, and >60, indicating that 48% of respondents were age 31 – 45 years old.

2. Number of elephants and KAP: similarly, the range of 2-5 elephants is too wide. Recategorizing this variable could provide more nuanced insights.

- Response in *Table 1 (Page 13):* recategorized the number of elephants under care to four groups, including 1, 2-3, 4-5, and > 5, indicating that 42% of respondents cared for 2-3 elephants.

3. Experience level and KAP: recategorizing the experience variable and conducting correlation analysis could reveal valuable relationship.

- Response in *Table 1 (Page 13):* recategorized the experiences with elephants to eight groups, including ≤ 5, 6-10, 11-20, 21-30, 31-40, 41-50, 51-60, and >60, indicating that most of the respondents had 11-40 years of experiences with elephants.
- Response in *Page 19, line 378-381*: However, there was no significant difference between the experience of mahouts toward the knowledge, attitude, and practices of mahouts even recategorized.

**Reviewer3:**

- Topic: EEHV is not generally recognized by readers, mostly by elephant people only, thus please add the full name with abbreviation in bracket
- *Response in Title (Page 1*): changed to “Mahout’s Perceptions and Practices in Managing Elephant Endotheliotropic Herpesvirus (EEHV) infection Among Captive Asian Elephants in Surin province, Thailand.”
- Introduction: As there were reports and epidemiology of the EEHV occurrence in Thailand (Boonprasert et al., 2019, Yun et al., 2021), please mentioned or showed the previous case number of EEHV cases in the elephant kingdom project
  - Response in *Page 5, line 84-85*: added “Addition, a total of 11 confirmed EEHV cases was reported in Surin with 66.7% fatality rate from 2006 – 2019 (Boonprasert et al., 2019, Yun et al., 2021)” in the introduction part.
  - Response in *Page 23, line 445-449*: added “Although eleven EEHV clinical cases had been confirmed in this area previously, none of the EEHV clinical cases were recorded during study period. However, twelve and two elephant claves were tested positive for EEHV without developing clinical signs in 2020 and 2021, respectively” in the discussion part.
- Line 30: please add “(KAP score)”
  - Response in *Page 2, line 32*: added “(KAP score)”
- Line 106: Knowledge, attitude, and practices should be changed as KAP only, as it was repeated
  - Response in *Page 6, line 124*: changed to “KAP”.
- Line 174: 32 should be changed to 31
  - Response in *Page 10, line 207*: changed to “31”.
- Line 118-119, 191: please use only “EKP”, as the full name mentioned in line 62
  - Response in *Page 7, line 140 and Page 11 line 232*: changed to “EKP”.
- Results: the score of each KAP categories was very low (knowledge 5.71 from 30, attitude 9.67 from 17, practice 2.3 from 10). Please discuss more in the discussion part
- What is the interpretation of each category?
- Response in *Page 28, line 539- 549:* the low scores in knowledge may attribute from the beliefs in long-established cultural practices which influence the acceptance a new knowledge/information
- Response in *Page 25-26, line 479-496:* Low attitude may be attributed from the lack of understanding the disease and the previous personal experience leading to affect some negative attitude.
- Response in *Page 28, line 548-551:* Low practice may be attributed from the low knowledge which shows the significant relation to low practice score and the deeply rooted belief in traditional elephant rearing.
- Is that much differ from the KAP score in other previous studies of other infectious disease e.g. COVID-19?
  - Answer: there is no evidence of using KAP score in other infectious diseases in this group of respondents (either mahouts or villagers in this study areas), thus the authors can not discuss about the differentiation. However, the study on KAP score on the management of elephant Laos was compared and described in the discussion (*Page 23, line 430-436).*
- Line 213: “ranging from 0 to 11” and refer to Table2, which showed the range of “0-17”. I am not sure which one is correct?
  - Response in *Page 14, line 272*: changed to “0-17”.
- Line 273-278: the authors showed the results of transportation with cleanliness. Please discuss more in the discussion part.
  - Response in *Page 27, line 516-527*: added data regarding to the suggested cleaning.
- Line 386-389: the authors mentioned “recommendation to apply the KAP survey in other parts of the country” in the abstract, but no mentioned in the end of the discussion part or at the conclusion part. Please add this part.
- Response in *Page 29, line 565-567*: added “Furthermore, it is recommended to apply the KAP survey in different elephant-handling cultures to understand their traditional practices and explore ways to integrate modern knowledge into their communities.”
- Table 2 and Table 3: There were five columns in Table 2 and 4, and four columns in Table 3. The different was the “range” column; therefore, this should be consistent.
  - Response in *Table 3 (Page 16), line 312*: added “range” column in Table 3.
- General comment: the author used ‘calf elephant’. In my opinion, it’s better to use ‘elephant calf’, which is similar to several article.
  - Changed “calf elephant” to “elephant calf” throughout the manuscript, as reviewer suggested.

The revised manuscript has not been published elsewhere, accepted for publication elsewhere, nor is it under editorial review for publication elsewhere. The revised manuscript has been read and approved by all listed authors.

Sincerely yours,

Supaphen Sripiboon (Corresponding author)
